# Supplementary material for: Optimization of DNA Fragmentation Techniques to Maximize Coverage Uniformity of Clinically Relevant Genes Using Whole Genome Sequencing
Source: Diagnostics (Basel). 2025 Sep 10;15(18):2294. doi: 10.3390/diagnostics15182294 (PMC12468493; doi:10.3390/diagnostics15182294)
Supplement: Supplementary file 1 [file diagnostics-15-02294-s001.zip › diagnostics-3817343-supplementary.pdf]

Supplementary Information

Table S1. TSO500 genes. List of the 504 genes that were used to analyze coverage uniformity across the library kits and sample types. These genes are used in the CLC Genomics TSO500 DNA analysis workflow, excluding 23 genes on the X chromosome.

ABL1	*CDK12*	*ERG*	*GNAQ*	*KEAP1*	*NFE2L2*	*PRKDC*	*SMO*
ABL2	*CDK4*	*ERRFI1*	*GNAS*	*KEL*	*NFKBIA*	*PRKG1*	*SNCAIP*
ACVR1	*CDK6*	*ESR1*	*GPR124*	*KIF5B*	*NKX2-1*	*PRSS8*	*SOCS1*
ACVR1B	*CDK8*	*ETS1*	*GPS2*	*KIT*	*NKX3-1*	*PTCH1*	*SOX10*
AKT1	*CDKN1A*	*ETV1*	*GREM1*	*KLF4*	*NOTCH1*	*PTEN*	*SOX17*
AKT2	*CDKN1B*	*ETV4*	*GRIN2A*	*KLHL6*	*NOTCH2*	*PTPN11*	*SOX2*
AKT3	*CDKN2A*	*ETV5*	*GRM3*	*KMT2A*	*NOTCH3*	*PTPRD*	*SOX9*
ALK	*CDKN2B*	*ETV6*	*GSK3B*	*KMT2B*	*NOTCH4*	*PTPRS*	*SPEN*
ALOX12B	*CDKN2C*	*EWSR1*	*H3F3A*	*KMT2C*	*NPM1*	*PTPRT*	*SPOP*
ANKRD11	*CEBPA*	*EZH2*	*H3F3B*	*KMT2D*	*NRAS*	*QKI*	*SPTA1*
ANKRD26	*CENPA*	*FAM175A*	*H3F3C*	*KRAS*	*NRG1*	*RAB35*	*SRC*
APC	*CHD2*	*FAM46C*	*HGF*	*LAMP1*	*NSD1*	*RAC1*	*SRSF2*
ARFRP1	*CHD4*	*FANCA*	*HIST1H1C*	*LATS1*	*NTRK1*	*RAD21*	*STAG1*
ARID1A	*CHEK1*	*FANCC*	*HIST1H2BD*	*LATS2*	*NTRK2*	*RAD50*	*STAT3*
ARID1B	*CHEK2*	*FANCD2*	*HIST1H3A*	*LMO1*	*NTRK3*	*RAD51*	*STAT4*
ARID2	*CIC*	*FANCE*	*HIST1H3B*	*LRP1B*	*NUP93*	*RAD51B*	*STAT5A*
ARID5B	*CREBBP*	*FANCF*	*HIST1H3C*	*LYN*	*NUTM1*	*RAD51C*	*STAT5B*
ASXL1	*CRKL*	*FANCG*	*HIST1H3D*	*LZTR1*	*PAK1*	*RAD51D*	*STK11*
ASXL2	*CSF1R*	*FANCI*	*HIST1H3E*	*MAGI2*	*PAK7*	*RAD52*	*STK40*
ATM	*CSF3R*	*FANCL*	*HIST1H3F*	*MALT1*	*PALB2*	*RAD54L*	*STT3A*
ATR	*CSNK1A1*	*FAS*	*HIST1H3G*	*MAP2K1*	*PARK2*	*RAF1*	*SUFU*
AURKA	*CTCF*	*FAT1*	*HIST1H3H*	*MAP2K2*	*PARP1*	*RANBP2*	*SUZ12*
AURKB	*CTLA4*	*FBXW7*	*HIST1H3I*	*MAP2K4*	*PAX3*	*RARA*	*SYK*
AXIN1	*CTNNA1*	*FGF1*	*HIST1H3J*	*MAP3K1*	*PAX5*	*RASA1*	*TBX3*
AXIN2	*CTNNB1*	*FGF10*	*HIST2H3A*	*MAP3K13*	*PAX7*	*RB1*	*TCEB1*
AXL	*CUL3*	*FGF14*	*HIST2H3C*	*MAP3K14*	*PAX8*	*RECQL4*	*TCF3*
B2M	*CUX1*	*FGF19*	*HIST2H3D*	*MAP3K4*	*PBRM1*	*REEP5*	*TCF7L2*
BAP1	*CXCR4*	*FGF2*	*HIST3H3*	*MAPK1*	*PDCD1*	*REL*	*TERC*
BARD1	*CYLD*	*FGF23*	*HNF1A*	*MAPK3*	*PDCD1LG2*	*RET*	*TERT*
BBC3	*DAXX*	*FGF3*	*HNRNPK*	*MAX*	*PDGFRA*	*RFWD2*	*TET1*
BCL10	*DCC*	*FGF4*	*HOXB13*	*MCL1*	*PDGFRB*	*RHEB*	*TET2*
BCL2	*DCUN1D1*	*FGF5*	*HRAS*	*MDC1*	*PDK1*	*RHOA*	*TFRC*
BCL2L1	*DDR2*	*FGF6*	*HSD3B1*	*MDM2*	*PDPK1*	*RICTOR*	*TGFBR1*
BCL2L11	*DDX41*	*FGF7*	*HSP90AA1*	*MDM4*	*PGR*	*RIT1*	*TGFBR2*
BCL2L2	*DHX15*	*FGF8*	*HSPH1*	*MEF2B*	*PHOX2B*	*RNF43*	*TMEM127*
BCL6	*DICER1*	*FGF9*	*ICOSLG*	*MEN1*	*PIK3C2B*	*ROS1*	*TMPRSS2*
BCR	*DIS3*	*FGFR1*	*ID3*	*MET*	*PIK3C2G*	*RPS6KA4*	*TNFAIP3*
BIRC3	*DNAJB1*	*FGFR2*	*IDH1*	*MGA*	*PIK3C3*	*RPS6KB1*	*TNFRSF14*
BLM	*DNMT1*	*FGFR3*	*IDH2*	*MITF*	*PIK3CA*	*RPS6KB2*	*TOP1*
BMPR1A	*DNMT3A*	*FGFR4*	*IFNGR1*	*MLH1*	*PIK3CB*	*RPTOR*	*TOP2A*
BRAF	*DNMT3B*	*FH*	*IGF1*	*MLLT3*	*PIK3CD*	*RUNX1*	*TP53*
BRCA1	*DOT1L*	*FLCN*	*IGF1R*	*MPL*	*PIK3CG*	*RUNX1T1*	*TP63*
BRCA2	*E2F3*	*FLI1*	*IGF2*	*MRE11A*	*PIK3R1*	*RYBP*	*TRAF2*
BRD4	*EED*	*FLT1*	*IKBKE*	*MSH2*	*PIK3R2*	*SDHA*	*TRAF7*
BRIP1	*EGFL7*	*FLT3*	*IKZF1*	*MSH3*	*PIK3R3*	*SDHAF2*	*TSC1*

BTG1	*EGFR*	*FLT4*	*IL10*	*MSH6*	*PIM1*	*SDHB*	*TSC2*
C11orf30	*EIF4A2*	*FOXA1*	*IL7R*	*MST1*	*PLCG2*	*SDHC*	*TSHR*
CALR	*EIF4E*	*FOXL2*	*INHA*	*MST1R*	*PLK2*	*SDHD*	*U2AF1*
CARD11	*EML4*	*FOXO1*	*INHBA*	*MTOR*	*PMAIP1*	*SETBP1*	*VEGFA*
CASP8	*EP300*	*FOXP1*	*INPP4A*	*MUTYH*	*PMS1*	*SETD2*	*VHL*
CBFB	*EPCAM*	*FRS2*	*INPP4B*	*MYB*	*PMS2*	*SF3B1*	*VTCN1*
CBL	*EPHA3*	*FUBP1*	*INSR*	*MYC*	*PNRC1*	*SH2B3*	*WISP3*
CCND1	*EPHA5*	*FYN*	*IRF2*	*MYCL*	*POLD1*	*SHQ1*	*WT1*
CCND2	*EPHA7*	*GABRA6*	*IRF4*	*MYCN*	*POLE*	*SLC7A8*	*XPO1*
CCND3	*EPHB1*	*GATA2*	*IRS1*	*MYD88*	*PPARG*	*SLIT2*	*XRCC2*
CCNE1	*ERBB2*	*GATA3*	*IRS2*	*MYOD1*	*PPM1D*	*SLX4*	*YAP1*
CD274	*ERBB3*	*GATA4*	*JAK1*	*NAB2*	*PPP2R1A*	*SMAD2*	*YES1*
CD276	*ERBB4*	*GATA6*	*JAK2*	*NBN*	*PPP2R2A*	*SMAD3*	*ZBTB2*
CD74	*ERCC1*	*GEN1*	*JAK3*	*NCOA3*	*PPP6C*	*SMAD4*	*ZBTB7A*
CD79A	*ERCC2*	*GID4*	*JUN*	*NCOR1*	*PRDM1*	*SMARCA4*	*ZFHX3*
CD79B	*ERCC3*	*GLI1*	*KAT6A*	*NEGR1*	*PREX2*	*SMARCB1*	*ZNF2*
CDC73	*ERCC4*	*GNA11*	*KDM5A*	*NF1*	*PRKAR1A*	*SMARCD1*	*ZNF217*
CDH1	*ERCC5*	*GNA13*	*KDR*	*NF2*	*PRKCI*	*SMC3*	*ZNF703*

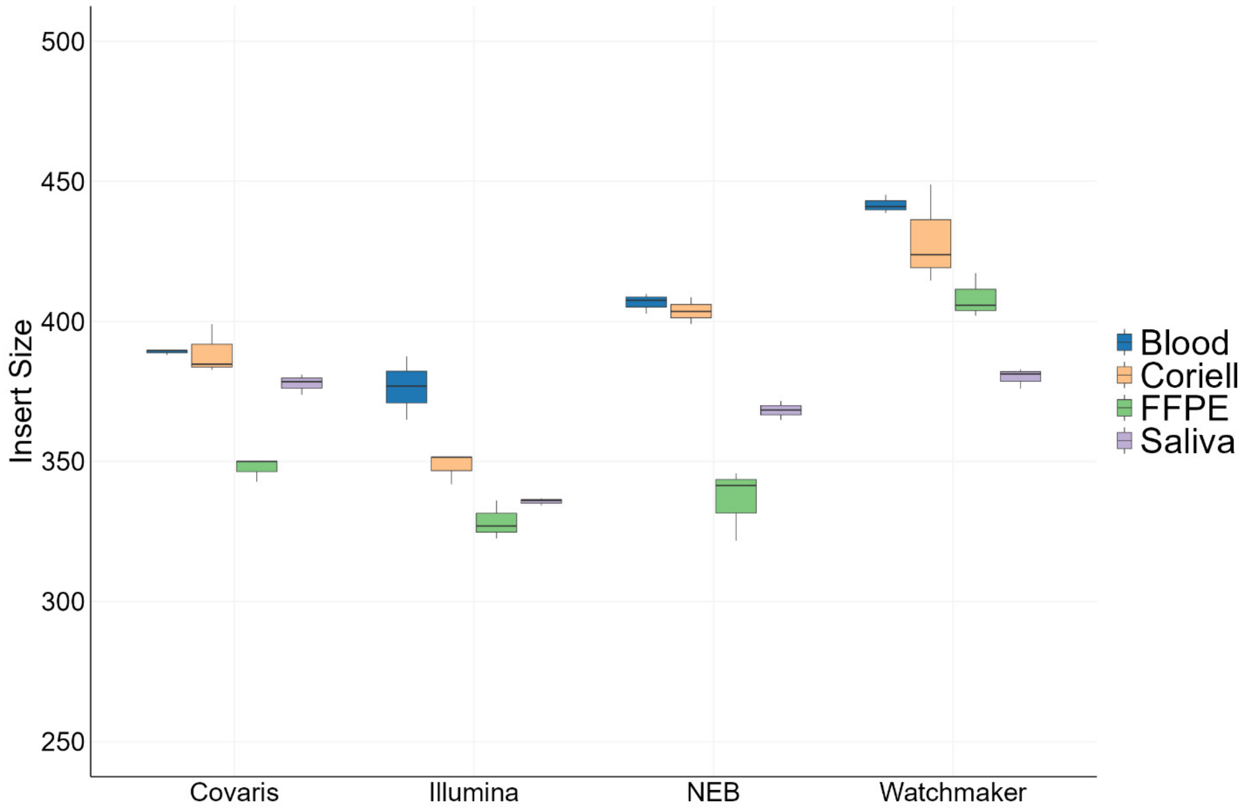

Figure S1. Sequenced Average Insert Sizes. Boxplots of average insert sizes across the different library kits and sample types.

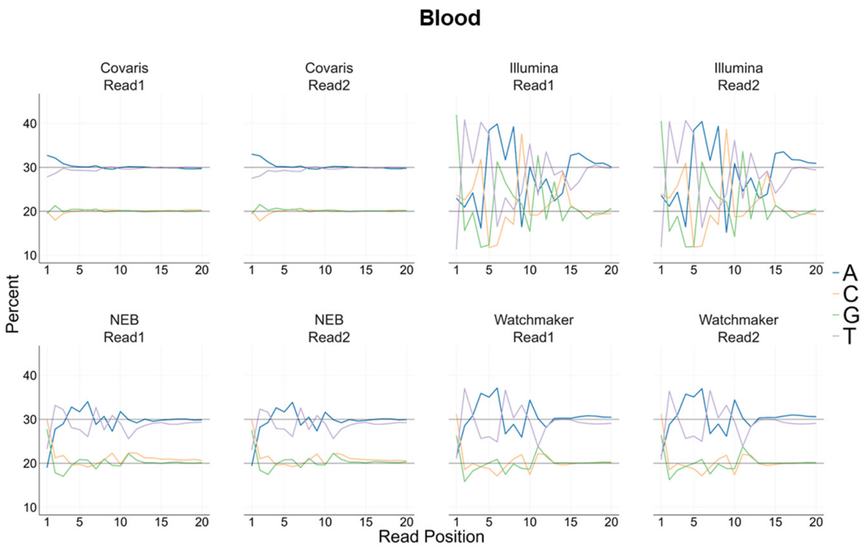
Blood
Covaris Read1
Covaris Read2
Illumina Read1
Illumina Read2
NEB Read1
NEB Read2
Watchmaker Read1
Watchmaker Read2
Percent
Read Position
A
C
G
T

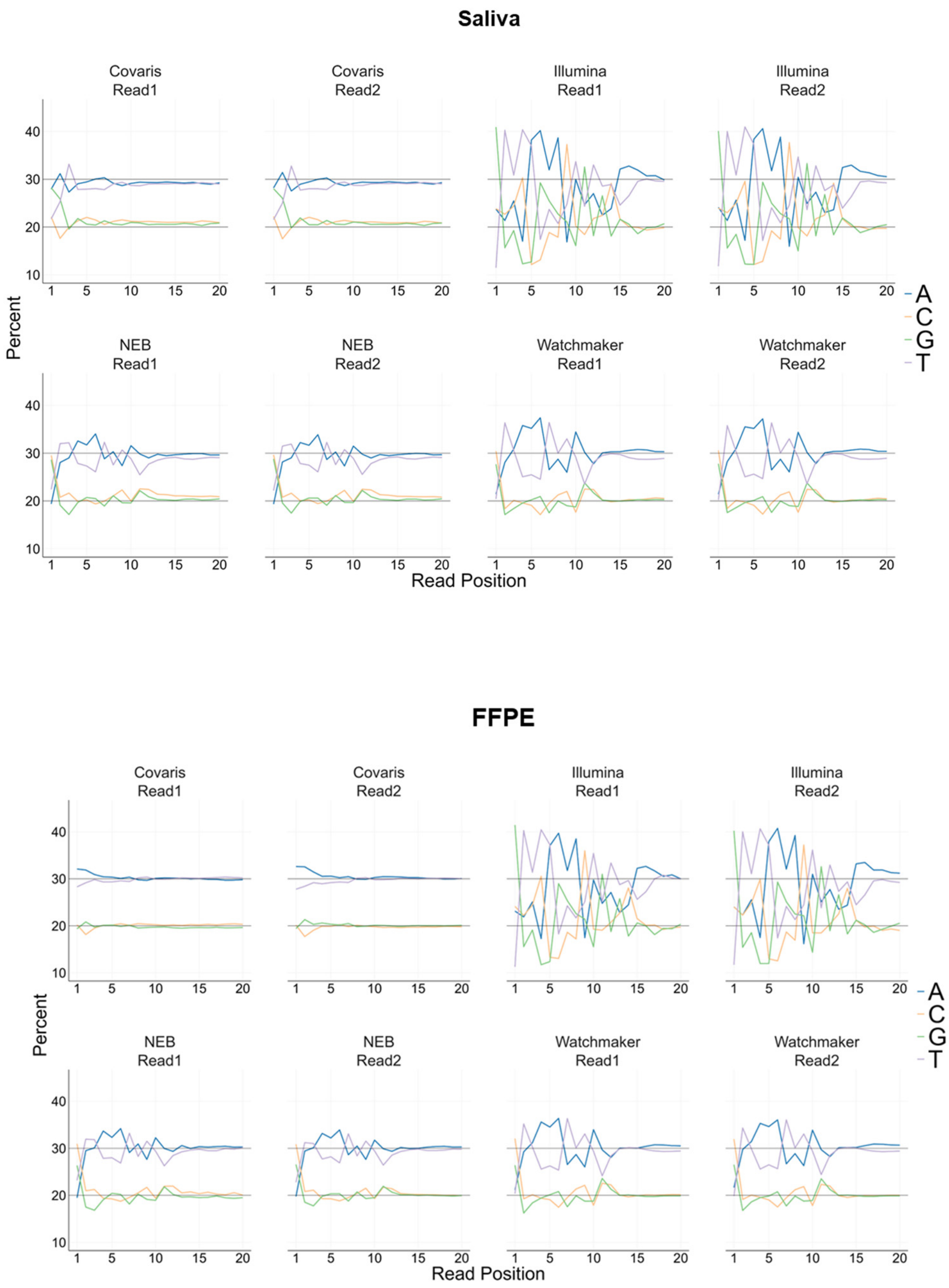

Figure S2. Base Composition of first 20 sequenced reads with blood, saliva and FFPE DNA samples. Line plots show the percentage of base composition in the first 20 bases of Read 1 and Read 2 for each library prep kit. Each line represents a different nucleotide base.

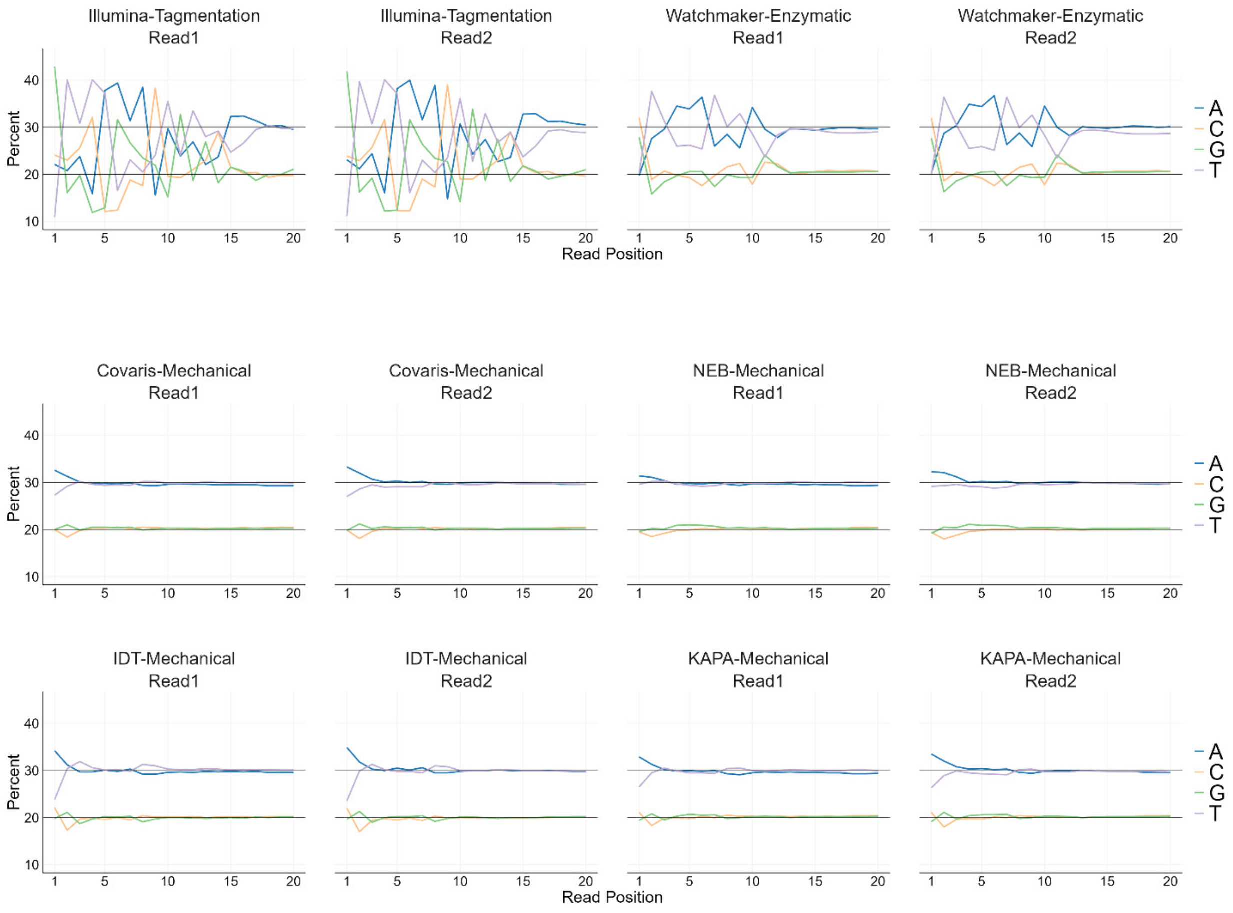

Figure S3. Base composition of Coriell DNA between non-mechanical and mechanical fragmentation. The library kits using tagmentation or enzymatic fragmentation show base composition bias for all DNA bases. The library kits utilizing Covaris mechanical fragmentation from four different vendors (Covaris, IDT, KAPA, and NEB) demonstrate minimal bias and a base composition consistent with the human genome. Additionally, repeat testing with a few enzymatic fragmentation options (Illumina and Watchmaker) confirmed persistent bias at the point of fragmentation.

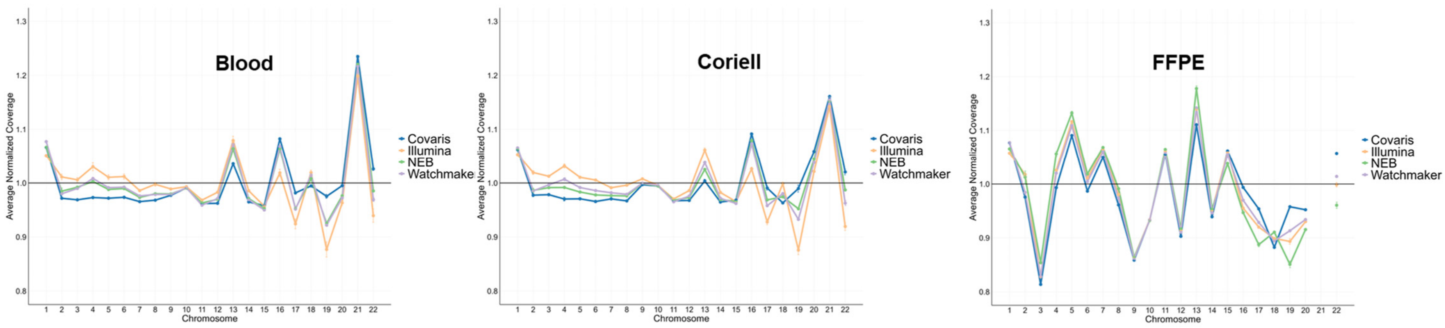

Figure S4. Chromosomal coverage distribution normalized by total coverage. Line plot of normalized coverage across the autosomal chromosomes for blood, Coriell and FFPE samples, with each line representing a different library kit. X-axis are the 22 autosomal chromosomes.

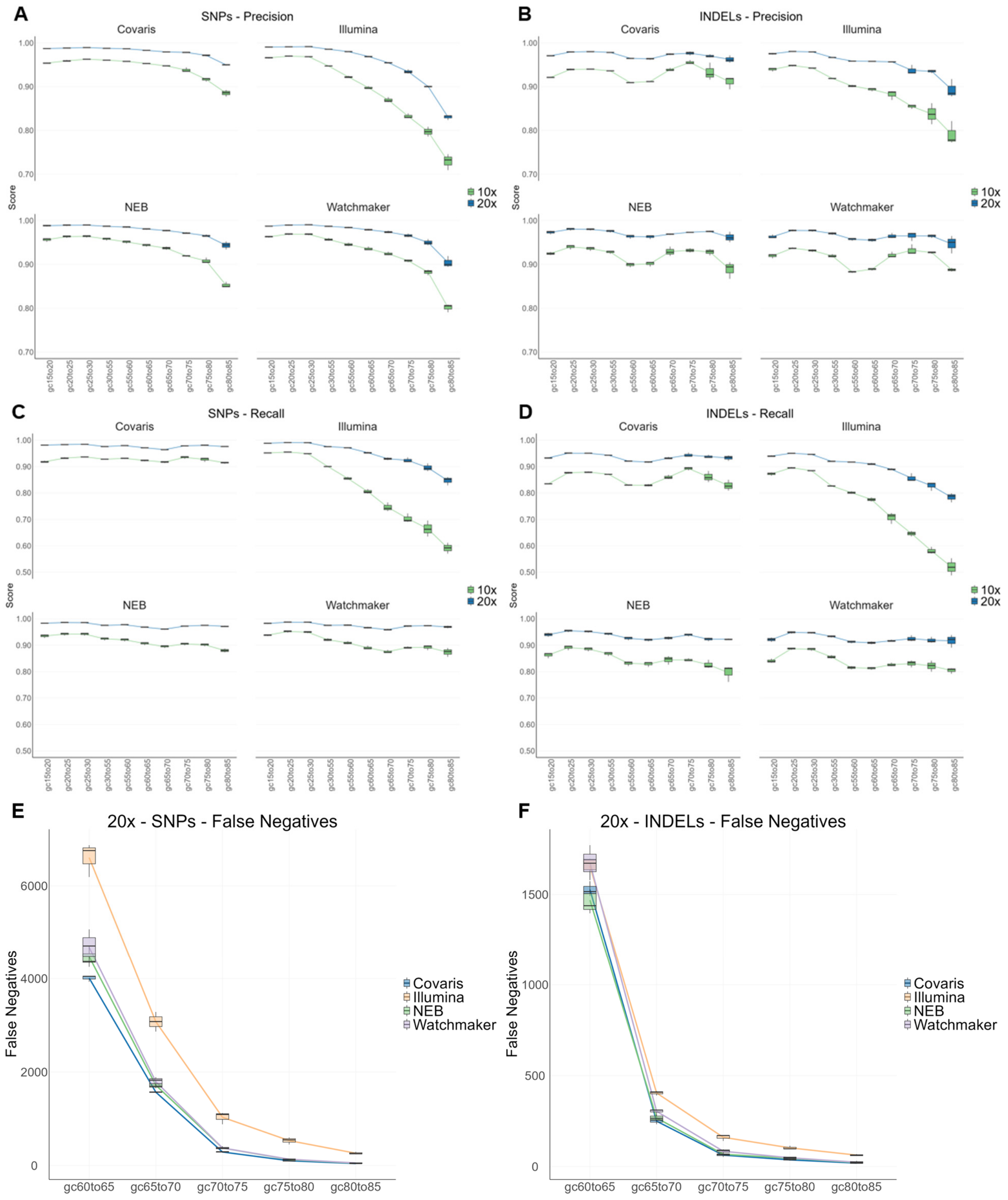
A
SNPs - Precision
B
INDELs - Precision
C
SNPs - Recall
D
INDELs - Recall
Covaris
Illumina
NEB
Watchmaker
Score
10x
20x
E
20x - SNPs - False Negatives
F
20x - INDELs - False Negatives
False Negatives
gc60to65
gc65to70
gc70to75
gc75to80
gc80to85
Covaris
Illumina
NEB
Watchmaker

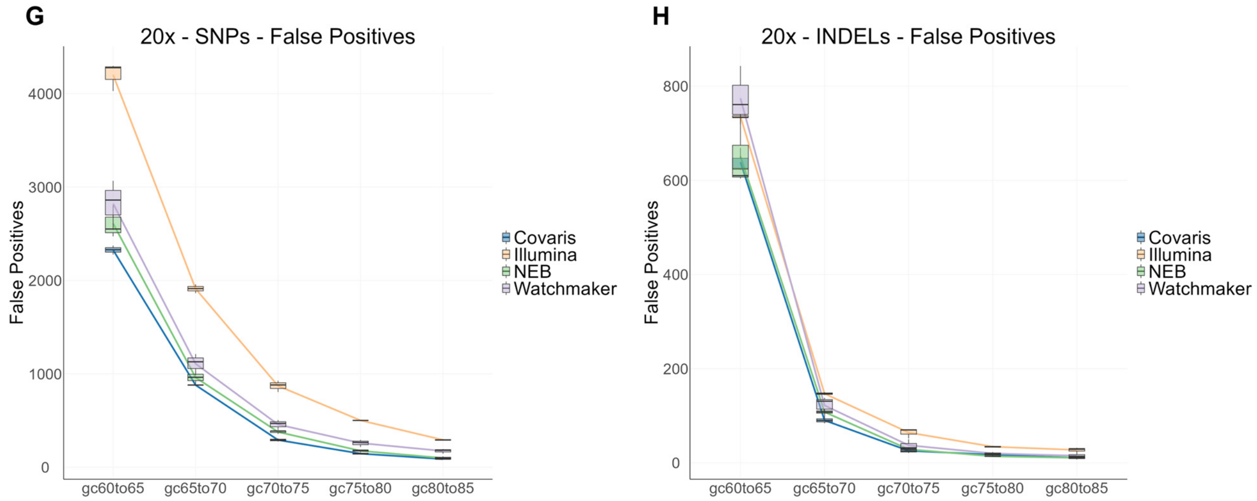

Figure S5. Variant performance for NA12878 across the GIAB GC content stratification regions as described in Figure 7. **(A-B)** Precision data for SNPs and Indels, **(C-D)** Recall data for SNPs and Indels, **(E-F)** For 20x coverage data only, False Negatives in high GC content regions (60 – 85 % GC) and **(G-H)** False Positives in high GC content regions (60 – 85 % GC).

Table S2. Summary of NA12878 variant calling performance metrics within TSO500 regions across library preparation workflows. The table summarizes the number of false negatives (FN), false positives (FP), and true positives (TP) for single nucleotide variants (SNVs) detected in libraries prepared using Covaris, Illumina, NEB, and Watchmaker workflows.

**Variant Type**	**Library Kit**	**Average Number Variants (10x)**	**Standard of Deviation Variants (10x)**	**Average of Number Variants (20x)**	**Standard of Deviation of Variants (20x)**
False Negatives	Covaris	27	5	10	4
	Illumina	73	9	14	4
	NEB	33	14	10	2
	Watchmaker	41	10	9	6
False Positives	Covaris	38	2	19	5
	Illumina	62	6	23	4
	NEB	36	7	14	3
	Watchmaker	50	2	21	3
True Positives	Covaris	522	5	539	4
	Illumina	476	9	535	4
	NEB	516	14	539	2
	Watchmaker	508	10	540	6
